# Associations in time between salivary cortisol and emotions in depressed patients and controls

**DOI:** 10.1192/j.eurpsy.2023.390

**Published:** 2023-07-19

**Authors:** A.-S. Koning, S. H. Booij, O. C. Meijer, H. Riese, E. J. Giltay

**Affiliations:** ^1^Endocrinology, Leiden University Medical Center, Leiden; ^2^Psychiatry, University Medical Center Groningen, Groningen; ^3^Psychiatry, Leiden University Medical Center, Leiden, Netherlands

## Abstract

**Introduction:**

Depression can be understood as a complex dynamic system, where depressive symptoms can directly affect each other. Knowledge on this symptom-symptom interaction is still scarce and is likely to differ between individuals. The hypothalamic-pituitary-adrenal (HPA) axis is often implicated in depression, with hypercortisolism and impaired glucocorticoid receptor-mediated feedback inhibition being commonly reported. High salivary cortisol levels may reflect a reaction to symptoms, or may rather be a cause, effectively ‘binding’ symptoms.

**Objectives:**

We aimed to analyze the temporal interplay between salivary cortisol and emotions in depressed patients and controls by using the novel Dynamic Time Warp analysis (DTW) approach.

**Methods:**

The ‘Mood and movement in daily life’ (MOOVD) study consisted of 30 pair-matched (15 depressed and 15 control) participants. Salivary cortisol was collected three times a day for 30 days, resulting in 90 measurements per individual. At the same moments, participants completed questionnaires on an electronic diary, which included different momentary positive (PA) and negative (NA) affect items. The dynamic interplay between salivary cortisol and affect were analyzed by DTW, which extends momentarily associations to include one earlier and one later time point, in both undirected and directed analyses.

**Results:**

Individual networks differed substantially within groups. At the group level, undirected and directed network analyses showed differences between depressed patients and controls. In undirected analysis, connectivity of PA items was comparable between depressed patients and controls, but the NA items showed a less dense network in depressed patients. Directed DTW analyses indicated (p = 0.07) that increases in salivary cortisol preceded that of some NA items (e.g., tiredness) in controls, but tended to follow upon NA item increase (e.g., not feeling appreciated) in depressed patients.

**Conclusions:**

At the group level, connectiveness between NA items was substantially weaker in depressed patients compared to controls. As in complex systems strong internal connectivity facilitates “critical transitions” to different states, this may reflect (or explain) the persistence of a chronically depressed state. We preliminary conclude that high salivary cortisol in depression may be a consequence of NA, rather than a cause. Replication of these first exploratory findings are needed.

**Disclosure of Interest:**

None Declared

